# Spotlight on ROS and *β*3-Adrenoreceptors Fighting in Cancer Cells

**DOI:** 10.1155/2019/6346529

**Published:** 2019-12-14

**Authors:** Maura Calvani, Angela Subbiani, Marina Vignoli, Claudio Favre

**Affiliations:** ^1^Oncohematology Unit, Department of Pediatric Oncology, A. Meyer Children's University Hospital, Florence 50139, Italy; ^2^Department of Health Sciences, University of Florence, Florence 50139, Italy

## Abstract

The role of ROS and RNS is a long-standing debate in cancer. Increasing the concentration of ROS reaching the toxic threshold can be an effective strategy for the reduction of tumor cell viability. On the other hand, cancer cells, by maintaining intracellular ROS concentration at an intermediate level called “mild oxidative stress,” promote the activation of signaling that favors tumor progression by increasing cell viability and dangerous tumor phenotype. Many chemotherapeutic treatments induce cell death by rising intracellular ROS concentration. The persistent drug stimulation leads tumor cells to simulate a process called hormesis by which cancer cells exhibit a biphasic response to exposure to drugs used. After a first strong response to a low dose of chemotherapeutic agent, cancer cells start to decrease the response even if high doses of drugs were used. In this framework, *β*3-adrenoreceptors (*β*3-ARs) fit with an emerging antioxidant role in cancer. *β*3-ARs are involved in tumor proliferation, angiogenesis, metastasis, and immune tolerance. Its inhibition, by the selective *β*3-ARs antagonist (SR59230A), leads cancer cells to increase ROS concentration thus inducing cell death and to decrease NO levels thus inhibiting angiogenesis. In this review, we report an overview on reactive oxygen biology in cancer cells focusing on *β*3-ARs as new players in the antioxidant pathway.

## 1. Reactive Oxygen Nitrogen Species

Reactive species or free radicals include reactive oxygen and nitrogen species collectively and are termed reactive oxygen nitrogen species (RONS).

Oxygen metabolism generates highly reactive molecules called reactive oxygen species (ROS). ROS production results in normal cellular metabolism. Free radical is defined as a molecule with unpaired electron in the outer shell such as superoxide anion (^·^O_2_^−^) or hydroxyl radicals (^·^OH) and nonradicals such as hydrogen peroxide (H_2_O_2_) [1]. Reactive nitrogen species (RNS) is the subclass of RONS that contain nitrogen compounds such as nitric oxide (^·^NO), peroxynitrite (^·^ONOO), and nitrogen dioxide (NO_2_) [2].

### 1.1. Reactive Oxygen Species

**Figure 1:**
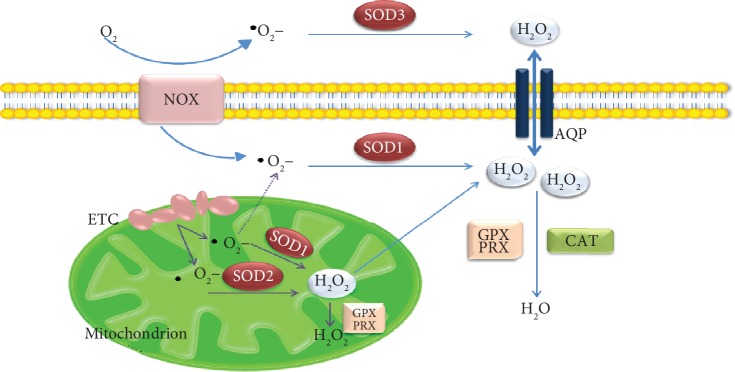
Schematic representation of ROS species within a cell.

Intracellular ROS are mainly generated in the mitochondria through activation of redox reactions catalyzed by specialized enzymes of electron transporter chain (ETC) where small amounts of oxygen are univalent and reduced into free radicals [3, 4], to produce cellular energy (adenosine triphosphate or ATP) [5] (Figure 1). Reduction of nicotinamide adenine dinucleotide (NADH) and flavin adenine dinucleotide (FADH2), which work as electron carriers, transfers the electron through the ETC. Subsequently, during oxidative phosphorylation, cytochrome c oxidase (COX or Complex IV) acts as the final acceptor of these electrons and catalyzed the tetravalent reduction of molecular oxygen (O_2_) into water (H_2_O) [6]. During this process, there is a nonfully efficient coupling between respiration and phosphorylation leading to proton and electron leaks. Ubisemiquinone, a component of Complex I in the mitochondria, represents the main site of electron leak leading to the generation of superoxide anions.

ROS could also be generated in response to growth factors, cytokines, or G protein-coupled receptor (GPCR) agonists through the activation of transmembrane enzymes called NADPH oxidase (nicotinamide adenine dinucleotide phosphate oxidase or NOX) family existing in different isoforms, widely distributed in different cell types [7]. Five NOX proteins have been evidenced and two further enzymes called Dual Oxidase (DUOX), containing a peroxidase-like domain [8, 9]. NOX are composed of six different subunits that interact to form an active enzyme complex. NOX catalyzes the production of a superoxide free radical by transferring one electron to oxygen from nicotinamide adenine dinucleotide phospate oxidase (NADPH). During this process, O_2_ is transported from the extracellular space to the cell interior and H^+^ is exported [10]. O_2_ has a short life, and it is dismutated fast in H_2_O_2_ spontaneously or enzymatically by the superoxide dismutase enzyme (SOD). Once generated, H_2_O_2_ preferentially enters the cell through specific plasma membrane aquaporin channels [11], activating different signaling.

At physiological condition, ROS have important roles in normal cellular functions, regulating different intercellular signaling pathways such as fighting against infection, facilitating normal maturation and fertilization in reproductive systems [1, 4, 12–15]. Depending on their concentration, ROS could trigger different intracellular pathways, thus leading to the activation of survival mechanisms or to the priming of the cell death program. ROS can reversibly oxidize target molecules such as cysteine (Cys) residues of phosphatases, increasing the level of protein phosphorylation. The oxidation of critical thiols is centrally involved in the transmission of a redox signal initiated by a protein modification of some amino acid side chains, such as Cys, methionine (Met), proline (Pro), histidine (His), and tryptophan (Trp) [16].

Redox homeostasis is maintained by the induction of a cell signaling cascade that controls ROS production and scavenging ability that efficiently maintain a stable concentration [17]. ROS production higher than physiological conditions induces temporary expression of many antioxidant molecules [5]. If the ROS increase is quite small, the antioxidant response can be able to balance the augmentation of ROS levels and restore the equilibrium between ROS production and scavenging ability. On the other hand, persistent cellular high ROS levels can lead to pathological conditions such as diabetes, neurodegenerative disorder, and cancer onset [18]. The first defense of endogenous neutralization of ROS is represented by enzymes such as SOD, catalase (CAT), and glutathione peroxidase (GPx). The nonenzymatic protection consists of different compounds such as glutathione (GSH), vitamin A, vitamin C, vitamin E, zinc, and selenium [19]. Among the enzymatic antioxidants, SODs convert ^·^O_2_^−^ to less reactive H_2_O_2_, CAT reduces H_2_O_2_ to H_2_O and O_2_, and GPx eliminates H_2_O_2_ using reducing power derived from GSH. Other important defensive mechanisms and mediators of redox signaling are represented by the peroxiredoxin (Prx), the thioredoxin (Trx), and the glutathione/glutaredoxin systems (GSH/Grx) [20–27]. Many cytoprotective enzymes, in response to reactive chemical stress, are regulated primarily at the transcriptional level. This transcriptional response is mediated by elements termed ARE (antioxidant response elements), initially found in the promoters of genes encoding the two major detoxification enzymes, glutathione S-transferase A2 (GSTA2) and NADPH quinone oxidoreductase-1 (NQO1). ARE binding increases the synthesis of many ARE-dependent antioxidant enzymes in different cells, such as glutathione reductase (GR), GPx, glutaredoxin (Grx), thioredoxin reductase (TrxR), heme oxygenase-1 (HO-1), and peroxiredoxin 1 (Prx-1).

The intracellular antioxidant defense in response to elevation of ROS content is regulated also by the nuclear factor erythroid 2-related factor 2 (Nrf2)/Kelch-like ECH-associated protein 1 (Keap1) pathway [28]. Keap1 is a cytosolic protein rich in cysteine residues that inhibits Nrf2 function through its binding, if cysteine residues are oxidized as a consequence of a change in the electrophilic balance Nrf2 is released and activated. In this active form, Nrf2 translocates into the nucleus and binds ARE located within the gene regulatory regions thereby regulating the expression of many target genes [29]. The GSH system and Prx-1 are two mechanisms whose expression is activated by Nrf2. Prxs are a highly conserved family of peroxidases that reduce peroxides, with a conserved cysteine residue, serving as the site of oxidation by peroxides. The peroxidase activity of Prx towards hydroperoxides and peroxynitrite, produced as a result of normal cellular metabolism in the cytosol, is critical to protect cellular components from oxidative damage [30].

HO are enzymes involved in heme catabolism; in humans, three isoforms of heme oxygenase are known. HO-1 is a stress-induced isoform present throughout the body with the highest concentrations in the spleen, liver, and kidneys and catalyzes the reduction of heme to biliverdin (BV), carbon monoxide, and ferrous ions in the presence of NADPH and oxygen. Biliverdin is subsequently converted to bilirubin (BR) by biliverdin reductase (BVR). Both BV and BR function as ROS scavengers. HO-1 expression is induced by oxidative stress and plays several roles in oxidative balance; in particular related to vasculature and diabetes [31], indeed, HO-1 knockout mice exhibit high susceptibility to hypertension [32].

### 1.2. Nitric Oxide

NO is a labile molecule involved in various physiological functions including vasodilatation and control of blood pressure [33], neurotransmission [34], immune response [35], and smooth muscle relaxation [36]. NO is produced from the metabolism of the amino acid L-arginine by nitric oxide synthases (NOS). There are three different NOS isoforms that differ in localization, regulation, and catalytic properties. Neuronal NOS (nNOS or type I NOS) and endothelial NOS (eNOS or type III NOS) are constitutively expressed (cNOS), while the other isoform is inducible and thus termed inducible NOS (iNOS) [33]. All NOS enzymes are homodimers. Each monomer contains a C-terminal reductase domain (NOSred) that binds NADPH, flavin adenine dinucleotide (FAD), and flavin mononucleotide (FMN) and a N-terminal oxygenase domain (NOSoxy) that contains the binding site for the cofactors cytochrome P-450-type heme and (6R-)5,6,7,8-tetrahydrobiopterin (BH_4_) and for the substrate L-arginine. NOS contain a zinc tetrathiolate cluster which consists in a zinc ion coordinated to two Cys residues in a CysXXXXCys motif, involved in BH_4_ and L-arginine binding to facilitate NOS dimerization. Binding of NADPH to the NOSred domain induces the electron transfer from NADPH to the heme-containing oxygenase domain, via reduction of FAD and FMN, with lower capacity to reduce O_2_ to O_2_^−^. The binding between Ca^2+^ and calmodulin (CaM) induces a conformational change of the FMN subdomain in proximity of the heme-containing oxygenase domain, and the electron transfers from FMN to the heme of the opposite monomer, thus explaining why monomeric NOS enzymes are inactive. The oxygenase domain can also bind to BH_4_ and L-arginine which stabilize the dimeric form of NOS though the heme domain coupling and promote the efficient O_2_ reduction and NO production. NOS synthesize NO by two different oxidation steps. In the first step, NOS oxidize L-arginine to *N^ω^*-hydroxy-L-arginine (L-NOHA), and in the second step, the enzyme oxidizes L-NOHA to L-citrulline and NO. BH_4_ provides the stabilization of the charge by the recruitment of an electron from the heme iron with subsequent release of NO out of the active site. nNOS and eNOS can be activated by Ca^2+^ and CaM to generate and release a small amount of NO. iNOS, by contrast, is primarily regulated at the transcriptional level, and it is only induced when the cell is stimulated by immunological signals such as proinflammatory cytokines (i.e., tumor necrosis factor-*α*, interleukin-1*β*, and interferon-*γ*), bacterial lipopolysaccharide (LPS), or infection, generating a larger amount of NO to contrast pathogen invasion. nNOS is mostly localized in specific neurons of the central nervous system and has been implicated in synaptic plasticity, learning, memory, and neurogenesis. Besides brain tissues, nNOS has been found in the spinal cord, skeletal and cardiac myocytes, pancreatic islet cells, sympathetic ganglia and adrenal glands, parasympathetic ganglia, kidney macula densa cells, peripheral nitrergic nerves, and epithelial cells [37]. In the peripheral nervous system (PNS), NO acts as a neurotransmitter decreasing the tone of various types of smooth muscle including corpus cavernosum [38] and blood vessels [39]. Nevertheless, abnormal NO production, due to an overexpression or dysregulation of nNOS, leads to toxic effects that are associated with some human diseases such as septic shock, cardiac dysfunction, diabetes, and cancer [40]. eNOS is primarily expressed not only in endothelial cells [41] but also in platelets, cardiac myocytes, and neurons [39]. NO generated by eNOS is responsible to regulate cellular processes including vasodilation [42, 43] and inhibition of platelet aggregation and adhesion [44–46], control of vascular smooth muscle proliferation [47], angiogenesis [48, 49], activation of endothelial progenitor cells [50, 51], and inhibition of leucocyte adhesion and vascular inflammation [52, 53]. Besides Ca^2+^-CaM-mediated activation, eNOS can interact with several other proteins like heat shock protein 90 (Hsp90) and caveolar coat protein caveolin-1 (Cav-1) which activate and repress the enzyme activity respectively [54–56]. Moreover, eNOS can be activated and regulated by phosphorylation on serine (Ser) residues and, to a lesser degree, on tyrosine (Tyr) and threonine (Thr) residues. Phosphorylation of Ser1177 and dephosphorylation of Thr495 stimulate the increase of the intracellular concentration of Ca^2+^, resulting in eNOS activation [41, 57, 58], while Tyr phosphorylation is associated with a decrease in catalytic activity [55]. In particular, the involvement of Thr495-dephosphorylation in the uncoupling of eNOS has been reported and is due to an increase in ROS production and oxidative stress that lead to several human pathology such as atherosclerosis, ischemia, diabetes, and hypertension [57, 59]. ROS production in oxidative stress leads to the production of O_2_^−^ instead of NO with oxidation of BH_4_ [60, 61], depletion of L-arginine [62, 63], and S-glutathionylation of eNOS [64], resulting in NO reduction and increase of oxidative stress. iNOS is mostly expressed in macrophages to protect against pathogens; thus, it is critical for the inflammatory response and the innate immune system, but iNOS is also expressed potentially in any cells or tissues following stimulation [65]. The binding of NO to the iron in the catalytic sites of the enzymes and the interference with the DNA of target cells lead to the inhibition of enzymes involved in the principal physiological functions (i.e., citric acid cycle, DNA replication, and ETC) [66] and DNA fragmentation respectively [67, 68]. In addition to eNOS, iNOS activity can be modulated by mutations in amino acid residues. Arg375 mutation of BH_4_ in the NOSoxy domain and Phe831 and Leu832 in the NOSred domain result in the decrease of iNOS activity [69, 70]. Higher levels of NO produced by iNOS of macrophages can lead to cellular and tissue damage and septic shock which is characterized by vasodilatation, microvascular damage, and hypertension [71]. Recently, the presence of a mitochondrial NOS (mtNOS) has been evidenced, and it seems to be involved in the regulation of mitochondria and cellular functions in various type of tissues [72]. Mitochondria can produce and consume NO which stimulates mitochondrial biogenesis, through cGMP upregulation of transcriptional factors. In particular, NO inhibits mitochondrial respiration by binding the binuclear center of cytochrome *c* oxidoreductase (Complex III), leading to the inhibition of the enzyme activity with consequent inhibition of electron transfer and increase in O_2_^−^ production [73, 74].

## 2. ROS and Cancer

Increased levels of ROS have long been associated with different types of cancer, where they play a central role in cancer onset and progression [75]. Cancer cells maintain ROS level lightly higher than physiologic control, activating pathways that lead to cancer progression and metastases, and this state is called “mild oxidative” stress. The damages derived from oxidative stress include genome instability and consequently the increase of oncogenic mutations, loss of tumor suppressors, and changes in cancer cell metabolism [76]. At an advanced stage, cancer cell ROS-derived mutations lead to additional ROS generation by further supporting cancer progression. A hypoxic microenvironment has been described to play a central role in the increase of ROS in tumor, through the activation of the hypoxia-inducible factor 1 alpha (HIF-1*α*) and its target genes. In cancer cells, hypoxia leads to the activation of different genes that control cellular growth, survival, and proliferation. The increased rate of ROS production during cancer is mainly caused by a high metabolic rate in mitochondria, endoplasmic reticulum, and cell membrane. Hypoxia and metabolic changes lead to respiratory and mitochondria dysfunctions by impairing ETC, lowering coupling efficiency, and increasing electron leakage [18]. To maintain high energy levels, cancer cells switch their metabolism, enhancing glycolytic rate and lactic acid fermentation even in the presence of oxygen, thus increasing mitochondrial ROS production. During hypoxic exposure, many growth factors and cytokines are produced and the activation of the relative pathways leads to the upregulation of NOX and consequent increase of ROS, thus affecting survival pathways such as the PI3K/Akt, RAF/MEK/ERK1/2, and JAK/STAT pathways downstream of both growth factor stimulation and oncogene activity [77]. Vascular endothelial growth factor (VEGF), under hypoxia, activates NOX and mediates ROS production leading to amplification of pathways involved in endothelial cell proliferation and angiogenesis [78]. An upregulated expression of NOX proteins has been evidenced in many cancer cell types. [79]. ROS produced in transformed cells allow cancer cells to activate *c-Myc* oncogene [80], leading to cancer progression and metastasis. To prevent the increase of ROS and maintain redox balance, cancer cells increase their antioxidant ability; in this way, cancer cells maintain ROS at a mild level thus enhancing protumorigenic signaling pathways without inducing cancer cell death. Compared with normal cells, cancer cells have an altered redox environment, with a high rate of ROS production counter balanced by a high rate of ROS scavenging [81]. A recent evidence suggests that modifying the levels of ROS by the action of antioxidants or prooxidants could modulate tumor growth. A mild concentration of ROS yields cancer cells vulnerable to further ROS increase strongly dependent on their antioxidant defenses. On the other hand, exacerbate oxidative stress leads to cell apoptosis by direct or indirect ROS-mediated damage of proteins, lipids, and nucleic acids. Antioxidants are the first response of cells to neutralize ROS and survive. Many enzymes including CAT, SOD, GPx, and ETC enzymes are responsible for the transformation of free radicals into more stable and less damaging molecules. Many nonenzymatic antioxidants work as a ROS scavenger in cancer cells such as *β*-carotene and vitamins A, E, and C [82]. In addition, redox-sensitive transcription factors, including Nrf2 and HIF-1*α*, could also be activated to improve the action of antioxidants as well as to trigger the elevation of cell survival molecules such as the antiapoptotic protein Bcl-2 (B-cell lymphoma 2) and AKT (protein kinase B or PKB) [25]; the adaptive mechanism established by cancer cells in ROS response activates resistance to different cancer treatments. It has been reported that the increased antioxidant pathways driven by Nrf2 are involved in cancer progression [83–85]. However, the role of ROS in cancer is not one-sided. Notably, antioxidant enzyme-deficient cancer cells have less ability to form tumors in experimental mouse models. It has been reported that continuous antioxidant enzyme activity maintains metabolic activity and anchorage-independent growth in breast cancer cells. Thus, inhibiting antioxidant enzyme activity may be an effective strategy to enhance susceptibility to cell death in cancer cells [86]. CAT expression is modified in cancer cell lines that become resistant to chronic exposures to H_2_O_2_ [87, 88] or to certain chemotherapeutic agents such as doxorubicin [89–92]. Although mechanisms controlling CAT expression have been partially elucidated, the decreased CAT expression in cancer cells still remains an unanswered question. Chemotherapeutic agents lead to an exuberant increase of ROS content leading to induction of cellular damage and apoptosis of cancer cells by evoking toxicity. Depending on the tumor type, this goal can be reached by chemotherapy or radiation therapy [93]. Alkylating agent (alkyl sulfonates, ethyleneamines, and hydrazines), anthracyclines (doxorubicin and doxorubicin), platinum coordination complexes (cisplatin, carboplatin), podophyllin derivatives (etoposides), and camptothecins (irinotecan, topotecan) increase free radical production [94–97]. Many cytoprotective enzymes, in response to reactive chemical stress, are regulated primarily at the transcriptional level. This transcriptional response is mediated by ARE that control the expression of two major detoxification enzyme genes, GSTA2 and NQO1. Many cancer cells become resistant to chemotherapeutic agents by activating antioxidant response. This phenomenon is similar to hormesis, a process in which exposure to a low dose of a chemical agent is damaging at higher doses, induces an adaptive beneficial effect on the cell following an initial disruption in homeostasis [98].

## 3. NO and Cancer

Besides the signaling role in the neuronal, cardiovascular, and immune systems, NO is also involved in the pathogenesis and progression of different cancer types. A dual role of NO in cancer, depending on its localization and concentration, has been reported. At low concentration, NO modulates angiogenesis, cell cycle progression, apoptosis, invasion, and metastasis, while at higher levels, it acts as an antioncogenic agent promoting DNA damage, cytotoxic effects, oxidative stress, and apoptosis [99, 100]. The continuous production of NO by iNOS, is regulated by the tumor suppressor gene p53, which inhibits the enzyme through a negative feedback mechanism that is involved in cancer progression [99]. Several studies report the implication of iNOS in cancer. iNOS overexpression in prostate cancer cells and thyroid cancer demonstrates an anticancer effect due to cell death induction [101] and the inhibition of tumorigenesis [102], while in gastric cancers, hepatocellular carcinoma, melanoma, leukemia, and osteosarcoma, iNOS expression correlates with tumor progression and with the degree of malignancy [103]. Nevertheless, lower iNOS expression has been reported to promote pancreas cancerogenesis and liver metastasis [104]. NO can cause DNA damage by three different mechanisms: (i) inhibition of the DNA repair enzyme; (ii) direct DNA modifications, causing base deamination, nitration, and oxidation [105]; and (iii) formation of mutagenic species [106]. In particular, the inhibitory effect of NO on DNA repair represents the principal mechanism of inflammation in cancer [107]. The role of NO in angiogenesis is well-established, a critical event that promotes neovascularization and subsequently cell proliferation and tumor progression. NO stimulates the mediators of angiogenesis such as epidermal growth factor receptor (EGFR) [108], VEGF [109], and cyclooxygenase-2 (COX-2) signaling pathways that stimulate the synthesis of proangiogenic factors [110]. However, VEGF can stimulate eNOS itself by the activation of tyrosine kinase and protein kinase C (PKC) signaling [111], promoting angiogenesis in various cancer types [112, 113]. Moreover, ROS and RNS production during inflammation can cause DNA damage ad mutations that lead to cancerogenesis [114]. NO has been shown to play important role in modulating apoptosis through posttranslational modification, with proapoptotic or antiapoptotic effects, depending on NO concentration and the types of cells that are involved [115]. In general, lower concentration of NO can protect from apoptosis, while high concentration induces apoptosis [116]. The principal antiapoptotic effects exerted by NO in the mitochondrion are the inhibition of caspase-3 expression [117]. S-Nitrosylation of the Cys in the catalytic site of caspase-3 prevents the cleavage of the procaspase-3 to activated caspase-3, occluding the release of cytochrome *c*, resulting in apoptosis inhibition [118]. Conversely, S-nitrosylation on the heme iron of cytochrome *c* released in the cytosol induces caspase-3 activation and apoptosis [119]. NO secreted by iNOS activation induces Fas/CD95-tyrosine nitration (death receptor), preventing CD95-tyrosine phosphorylation leading to an antiapoptotic effect [120]. It has been reported that NO participates in cancer progression and invasion by inducing epithelial-to-mesenchymal transition (EMT). NO mediates the upregulation of E-cadherin expression, a cell adhesion molecule expressed in the early stage of EMT [121], and impairs the expression of matrix metalloproteinase 2 and matrix metalloproteinase 9 (MMP-2 and MMP-9) which have a central role in the remodeling of extracellular matrix and invasion [122]. Recently, NO has been reported to have a pivotal role in the immune system acting like an immunosuppressive messenger in the tumor microenvironment. NO may induce immunosuppression by decreasing T cell-mediated antitumoral responses [123, 124], promoting the recruitment and activation of myeloid-derived suppressor cells (MDSCs) [125] and inducing the acquisition of stem features by cancer cells as a mechanism to escape from the immune system [126].

## 4. *β*-Adrenergic Receptors

*β*-Adrenergic receptors (*β*-ARs) play an important role in a wide range of physiological responses mediated by catecholamines: adrenaline (A) and noradrenaline (NA). *β*-ARs belong to the GPCR family which consists of 7-membrane-spanning *α*-helical segments and an intracellular heterotrimeric G-protein complex (G_*αβγ*_). The receptor molecule also includes an extracellular N-terminal domain and a cytosolic C-terminal tail, which contains phosphorylation sites for GPCR kinases. Ligand binding induces a conformational change in the receptor that allows the intracellular part of the receptor to couple with a G-protein leading to the exchange of guanosine diphosphate (GDP) with guanosine triphosphate (GTP) and dissociation of the G-proteins into active G_*βγ*_ and G_*α*_ subunits. The downstream effects of GPCR activation are determined by the type of G_*α*_ subunit (G_*α*.S_, G_*α*.I_, G_*α*.q_, and G_*α*.12_) that is coupled to the receptor. There are three subtypes of *β*-ARs: *β*1-ARs, *β*2-ARs, and *β*3-ARs. *β*1-ARs are found primarily in the striatum cardiac muscle, juxtaglomerular apparatus, and adipocytes. *β*1-ARs activation lead to positive cardiac ionotropic and chronotropic effect antagonist, increasing heart rate and contractility, while in the kidneys, juxtaglomerular cells and adipocytes stimulate renin secretion and lipolysis respectively [127]. *β*2-ARs show a greater binding affinity to noradrenaline instead of adrenaline. *β*2-ARs are present on the gastrointestinal and bronchial smooth muscle cells, skeletal muscle cells, and liver. Activation of *β*2-ARs causes bronchodilation and general muscle relaxation, redirecting blood flow and mobilizing energy stores [128]. In addition, *β*2-ARs are expressed in multiple immune and nonimmune cells with a role in immunoregulation and immune response [129, 130]. *β*2-ARs activation is also associated with cancerogenesis [131–134] and EMT in melanoma, breast cancer, gastric cancer, prostate cancer, and colorectal adenocarcinoma [135–137]. *β*3-ARs are located primarily in the small intestine, adipose tissue (both brown and white), and vascular endothelium [138].

### 4.1. *β*3-Adrenergic Receptors

Since their discovery in 1989 [139], it seemed clear that *β*3-ARs have important physiological implication, including modulation of metabolism through the regulation of the adrenergic *β*-oxidation of fatty acids in adipocytes [140], vasodilation and relaxation to cardiac contractility [141], and relaxation of the smooth muscle cells of the detrusor muscle in the urinary bladder [142]. *β*3-ARs differ from *β*1- and *β*2-ARs for molecular structure and pharmacological profile, and this leads to support differential intracellular signaling. In fact, the serine (Ser) and threonine (Thr) residues at the C-terminus region in *β*1- and *β*2-ARs that are subjected to GPCR kinase- (GRK-) mediated regulation through phosphorylation and the consensus sequence for protein kinase A (PKA) are absent in *β*3-ARs [143]. Recently, several studies have reported that *β*3-ARs effects are either similar or opposite to *β*1- and *β*2-ARs stimulation due to the type of G_*α*_ subunits they are coupled to. In the brown adipose tissue (BAT), *β*3-ARs can activate G_*α*s_ (activator subunit) signaling [144] promoting lipolysis and thermogenesis by activation of the mitochondrial uncoupling protein 1 (UCP1) which uncoupled mitochondrial oxidative phosphorylation leading to a proton conductance pathway across the inner membrane and increasing the energy utilization [145]. Otherwise, in the ventricular myocardium, *β*3-ARs are coupled with G_*α*i_ (inhibitory subunit) proteins [146] which increases NO production through activation of eNOS leading to inhibition of cardiac contraction unlike *β*1- and *β*2-ARs [147]. High *β*3-ARs expression was also found in retinal diseases like retinopathy of prematurity (ROP) [148]. Hypoxia induces an increased release of NA and upregulation and activation of *β*3-ARs which promotes migration, invasion, and proliferation of human retinal cells [149], suggesting that the *β*3-ARs agonism induces proliferation of vessels in retinopathies through NO production and the accumulation of cyclic GMP (cGMP), which increases the release of VEGF [150]. Ischemic and hypoxic condition is also present in the tumor microenvironment and determines the release of NA from the sympathetic nervous system (SNS). As a result, the *β*3-ARs expressed by tumor cells and endothelial cells are activated, increasing the production of NO, which in turn modulates the release of VEGF, leading to final effects such as vasodilation, angiogenesis, inflammation, and metastasis [151].

Moreover, *β*3-ARs activation mediates relaxation of the smooth muscle of urinary bladder [142] and affects the function of the urothelium [152]. There are several reports that show two different mechanisms of *β*3-ARs activation. The first consists in the activation of adenylyl cyclase (AC) with the subsequent formation of cyclic adenosine monophosphate (cAMP) with a small involvement of this pathway in bladder relaxation. Supporting evidences showed that *β*3-ARs can also stimulate large conductance Ca^2+^-activated K^+^ (BKCa) channels in bladders, and this second mechanism is well established and can mediate relaxation of detrusor smooth muscle [153]. In the last few years, Mirabegron, the first *β*3-ARs agonist, has been developed. Mirabegron has been approved for its pronounced effect on reducing the bladder tone and the detrusor muscle *in vitro* in the OAB syndrome [154].

### 4.2. *β*3-Adrenergic Receptors and Cancer

**Figure 2:**
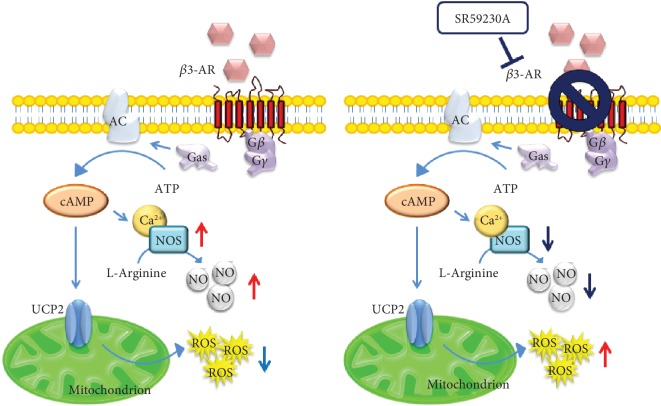
Schematic representation of *β*3-AR stimulation pathway and antioxidant function.

The relationship between *β*-ARs and cancer initiation and progression, including inflammation, angiogenesis, cell motility and trafficking, apoptosis/anoikis, cellular immune response, and EMT, has been well established. Over the years, several studies showed the overexpression of *β*-ARs across multiple cancer types and the clinical efficacy of pharmacological inhibition of the *β*-ARs with beta blockers as anticancer agents, supporting the evidence that *β*-blockers contribute to improve survival and decrease tumor proliferation and progression in multiple cancer types [155–158]. *β*1- and *β*2-ARs are expressed higher in non-small-cell lung cancer [159, 160], pancreatic cancer [161, 162], breast cancer [163, 164], ovarian carcinoma [165, 166], colorectal carcinoma [167], prostate cancer [168], and melanoma [169], while *β*3-ARs are detected in colon and breast cancer [155, 170], vascular tumors [171], human leukemia cells [172] and at very highly levels in melanoma [173]. In the last few years, numerous reports elucidated the involvement of *β*3-ARs in melanoma initiation and progression and the potential role of *β*3-ARs blocking to contrast tumor cells proliferation. In particular, it has been reported that the treatment with the specific *β*3-ARs antagonist SR59230A is effective in reducing melanoma angiogenesis and growth and in inducing apoptosis [174]. Besides the key role of *β*3-ARs in melanoma cancer cells, these receptors are also expressed in the melanoma microenviroment, such as cancer-associated fibroblast (CAF), endothelial cells, and macrophages, promoting secretion of proinflammatoy cytokines and *de novo* angiogenesis [173]. Calvani et al. also showed that *β*3-ARs are able to promote metabolic switch towards aerobic glycolysis by the induction of mitochondrial uncoupling protein 2 (UCP2), leading to proliferation of melanoma cells [175]. Recently, there has been a new role of *β*3-ARs reported in the regulation of melanoma immune-tolerance by increasing the number of cytotoxic immune cells, such as natural killer (NK) and CD8 T (CD8) cells, and by decreasing MDSC and regulatory T cells (T-reg) subpopulations [176]. Moreover, the protumoral role exerted by the *β*3-ARs has been recently confirmed in neuroblastoma (NB). In particular, *β*3-ARs blockade is able to switch from stemness features to a neuronal differentiation phenotype in NB cells, leading to a strong tumor growth reduction [177] (Figure 2).

## 5. Antioxidant Effects of the *β*3-ARs

The role of *β*3-ARs as antioxidants (Figure 2) has recently been evidenced in different studies. In the work of Yoshioka et al. [178], researchers showed that treatment of human astrocytoma U-251 MG cells with NA leads to an increase in the intracellular GSH concentration by inducing GCLc (Glutamate-Cysteine Ligase Catalytic Subunit) protein. In astrocytes, GSH synthesis is essential for the protection of neurons from oxidative damage induced by ROS. GSH acts either as an antioxidant by scavenging ROS species or as a cofactor for GPx that detoxifies a wide range of hydroperoxides. Results of the study suggested also that the NA-induced increase in GSH occurs through the stimulation of *β*3-ARs coupled to G_i/o_-protein but not to G_s_-protein. In another study, Hadi et al. [179] demonstrated the dual antioxidant capacity of *β*3-ARs: on the one hand, they reduce ROS production by direct inhibition of NOX; on the other hand, they specifically induce the expression of CAT, a major H_2_O_2_ scavenger. Therefore, they observed that *β*3-ARs stimulation has no effect on the expression of SOD-1 that would lead to H_2_O_2_ formation and its signaling, but rather it induces CAT expression. All the antioxidant effects mediated by *β*3-ARs depended on the activation of the transcription factor PPAR*γ* (Peroxisome Proliferator-Activated Receptor Gamma). Finally, they also confirmed a previously established link between ROS production and inflammatory induction: the authors observed that *β*3-ARs stimulation blocks the ROS-dependent NF*κ*B (nuclear factor kappa-light-chain-enhancer of activated B cell) pathway.

In a recent work, Calvani et al. [175] showed that *β*3-ARs are also expressed on the mitochondria of embryonic stem cells (ESC) and cancer stem cells (CSC), where their stimulation with the *β*3-ARs agonist BRL37344 induces an accelerated aerobic glycolysis (Warburg effect). This *β*3-AR-induced Warburg effect involves also UCP2, whose expression is inhibited by SR59230A that is implicated in mitochondrial ROS (mtROS) content modulation. The authors showed that SR59230A and Genipin (GN), a specific UCP2 inhibitor, increased mtROS content in both CSC and ESC treated with BRL37344, with a major increase in CSC. The relationship between ROS production and UCPs activity was revealed in 1997 from experiments where guanosine diphosphate (GDP), an UCP1 inhibitor, caused an increase of *Δψ* and ROS production [180], and later, it was demonstrated that superoxide directly activates UCPs resulting in a negative feedback controlling both ROS production and their levels [181]. Calvani et al. also showed, through functional analysis, that *β*3-ARs blockade in isolated mitochondria is able to decrease ATP production and to increase mtROS levels. These results confirm data already present in literature, where *β*3-ARs and UCP2 are indicated to have a strong antioxidant role [179, 182]. Moreover, these results clearly suggest that the *β*3-ARs/UCP2 axis promotes mitochondrial dormancy by inhibiting ATP production and mtROS content and leading both cell lines to increase aerobic glycolysis. In addition, *β*3-ARs antagonism promotes mitochondrial reactivation by inhibiting UCP2 activity and by increasing mtROS content. Thus, there is accumulating evidence supporting a direct link between mitochondria, oxidative stress, and cell death.

In another study, Pasha et al. [183] evaluated the effects of the treatment with Apigenin (a nutraceutical antioxidant) on cell lines derived from Ewing Sarcoma (ES). The authors observed that Apigenin induces partial ES cell death by inducing the activation of the apoptotic pathway without increasing mitochondrial ROS production that on the other hand is evidenced by administration of SR59230A. Apigenin inhibits the expression of antioxidant proteins such as superoxide dismutase 2 (SOD-2), CAT, Trx, sirtuin-1 (SIRT1), thioredoxin interacting protein (TXNIP), glutathione S-transferase Mu4 (GSTM4), and Nrf2 but increases the level of UCP2 and GSH which on the contrary are strongly inhibited by *β*3-ARs antagonism. The *β*3-ARs activity as antioxidants could be mediated by UCP2 protein expression that could control the redox homeostasis in ES cells. The redox homeostasis of cells is balanced by ROS generation and ROS quenching capacity. The disruption of UCP2 signaling together with the inhibition of *β*3-ARs activity causes an imbalanced elevation of ROS production within the cellular microenvironment that can lead to excessive oxidative stress resulting in massive cell death. Even if Apigenin inhibits antioxidant proteins, it works as the *β*3-ARs agonist, by avoiding the increase of mitochondrial ROS useful to achieve a massive cell death in ES cells; in this regard, administration of SR59230A improves the Apigenin effect on cell death.

*β*-Adrenergic signaling seems also to have a prominent role among the factors that may modulate the NO pathway and therefore affect tumor growth. NO contributes to tumor growth and metastasis by promoting migratory, invasive, and angiogenic properties of cancer cells in the majority of human tumors [184]. Different works have associated reduced NO levels with decreased growth of melanoma cells, while increased NO levels promote melanoma growth [185, 186]. Studies on melanoma cells suggest that NO derived from iNOS may stimulate proliferation as well as promote resistance to apoptosis [187] and reported a remarkable antitumor activity of iNOS inhibition that reduced melanoma growth and sensitize melanoma cells to chemotherapeutic agents [188]. In 2013, Dal Monte et al. hypothesized the involvement of iNOS in the antitumoral effects exerted by *β*3-ARs blockade in melanoma cells [174], and in a subsequent study, they confirm that the inhibitory effects of *β*3-ARs blockade on melanoma growth are mainly mediated by reduction of iNOS expression, resulting in a decreased activity in the NO pathway. *β*3-ARs blockade inhibits iNOS expression by reducing the basal nitrite production, while *β*3-ARs stimulation increases this production by activating iNOS expression. In addition, *β*3-ARs blockade effects are prevented by an NOS activator, and *β*3-ARs activation effects are prevented by an NOS inhibitor. These results show that NO exerts a protumorigenic function and iNOS-induced NO production is a key event in melanoma growth and that *β*3-ARs may regulate melanoma cell proliferation and survival through the NO pathway [189].

In a recent study evaluating the effect of nutraceutical antioxidant treatment of ES tumor cells A673, Calvani et al. observed that cells treated with curcumin, retinoic acid, 8-gingerol, and genistein exhibited reduced viability if compared with cells treated with capsaicin, ascorbic acid, formononetin, and flavon where the treatment did not affect cell viability. Moreover, cells treated with prosurvival antioxidants showed low levels of intracellular mitochondrial ROS, while the cells treated with antioxidants that are able to reduce cell viability are shown to be increased in mtROS levels. Interestingly, the treatments not affecting cell viability upregulated *β*3-ARs expression and the treatment that reduced cell viability strongly downregulated *β*3-ARs levels. These results identified the *β*3-ARs as the main regulators of the cellular response to oxidative stress under different micronutrient treatments. They function as ROS sensors in Ewing sarcoma cancer cells by driving or not antioxidant response and cell death. Since *β*3-ARs antagonism leads to massive cell death, inhibiting *β*3-ARs in these cells could dramatically increase the ROS levels by toxic threshold leading to cell death by inhibiting the antioxidant response of the cells [190].

## 6. Conclusion

**Figure 3:**
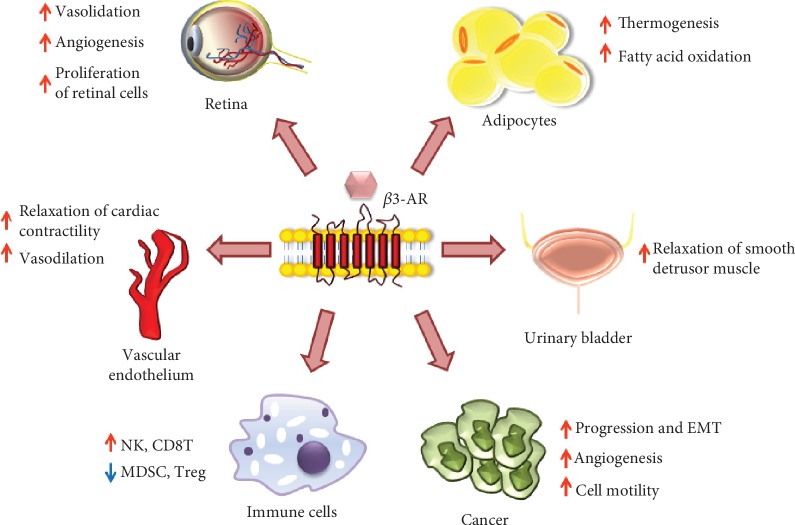
*β*3-AR role in different cells and tissues.

*In vitro* and *in vivo* data suggest that *β*3-ARs act as antioxidants in different cells by activating prosurvival factors and that their inhibition by a selective antagonist could be a new strategy to counteract tumor progression by elevating intracellular ROS concentration and activating apoptotic pathway (Figure 3). Moreover, *β*3-ARs antagonism inhibits NO production thus decreasing angiogenic switch in melanoma cells. In this review, it fully highlighted the new concept of *β*3-ARs as antioxidants and their ability to decrease ROS production and increase NO with multiple mechanisms (mitochondrial and NOX). Moreover, the concept that SR59230A strongly reduces cancer cell viability is highlighted, supporting the evidences that blocking *β*3-ARs function could represent a novel therapeutic strategy for the treatment of cancer by its ability to reduce antioxidant activity.
